# Co-circulation of seasonal influenza A(H1N1)pdm09, A(H3N2) and B/Victoria lineage viruses with further genetic diversification, EU/EEA, 2022/23 influenza season

**DOI:** 10.2807/1560-7917.ES.2024.29.39.2400020

**Published:** 2024-09-26

**Authors:** Eeva K Broberg, Olov Svartström, Maximilian Riess, Annette Kraus, Maja Vukovikj, Angeliki Melidou, Members of the European Reference Laboratory Network for Human Influenza (ERLI-Net), Collaborators, Amanda Bolt Botnen, Ramona Trebbien, Niina Ikonen, Erika Lindh, Vincent Enouf, Laurence Josset, Ralf Dürrwald, Marianne Wedde, Maria Exindari, Mary Emmanouil, Elaine Brabazon, Charlene Bennett, Simona Puzelli, Marzia Facchini, Ron Fouchier, Adam Meijer, Andreas Rohringer, Karoline Bragstad, Raquel Guiomar, Ana Paula Rodrigues, Mihaela Lazar, Rodica Popescu, Katarina Prosenc, Nataša Berginc, Francisco Pozo, Inmaculada Casas, Tove Samuelsson-Hagey, Neus Latorre-Margalef, Cornelia Adlhoch, Monica Galiano, Nicola Lewis

**Affiliations:** 1 European Centre for Disease Prevention and Control (ECDC), Stockholm, Sweden; 2 Members of the ERLI-Net that contributed virus characterisation data or were involved in weekly surveillance activities are listed under Collaborators and at the end of the article.

**Keywords:** influenza virus, characterisation, genetic, sequencing, Europe, surveillance

## Abstract

**Background:**

Influenza viruses can cause large seasonal epidemics with high healthcare impact and severity as they continually change their virological properties such as genetic makeup over time.

**Aim:**

We aimed to monitor the characteristics of circulating influenza viruses over the 2022/23 influenza season in the EU/EEA countries. In addition, we wanted to compare how closely the circulating viruses resemble the viral components selected for seasonal influenza vaccines, and whether the circulating viruses had acquired resistance to commonly used antiviral drugs.

**Methods:**

We performed a descriptive analysis of the influenza virus detections and characterisations reported by National Influenza Centres (NIC) from the 30 EU/EEA countries from week 40/2022 to week 39/2023 to The European Surveillance System (TESSy) as part of the Global Influenza Surveillance and Response System (GISRS).

**Results:**

In the EU/EEA countries, the 2022/23 influenza season was characterised by co-circulation of A(H1N1)pdm09, A(H3N2) and B/Victoria-lineage viruses. The genetic evolution of these viruses continued and clade 6B.1A.5a.2a of A(H1N1)pdm09, 3C.2a1b.2a.2b of A(H3N2) and V1A.3a.2 of B/Victoria viruses dominated. Influenza B/Yamagata-lineage viruses were not reported.

**Discussion:**

The World Health Organization (WHO) vaccine composition recommendation for the northern hemisphere 2023/24 season reflects the European virus evolution, with a change of the A(H1N1)pdm09 component, while keeping the A(H3N2) and B/Victoria-lineage components unchanged.

**Key public health message**

**What did you want to address in this study?**

Influenza viruses continually adapt their genetic sequences over time. To understand changes in genetic characteristics, we monitored influenza virus sequence changes in the 30 EU/EEA countries, compared circulating viruses to the viruses used to generate vaccines and determined whether they had acquired resistance.

**What have we learnt from this study?**

In the EU/EEA countries, during the 2022/23 influenza season, all seasonal influenza types and subtypes circulated. We detected all influenza virus (sub)types and observed continued genetic evolution. A(H1N1)pdm09 viruses diverged from the earlier influenza vaccine component but A(H3N2) and B/Victoria-lineage components matched well with the circulating viruses. Only 0.1% viruses were resistant to currently used antivirals.

**What are the implications of your findings for public health?**

Influenza virus surveillance data provide an important mechanism for monitoring the evolution of influenza viruses over the season. This remains a public health priority to ensure that influenza vaccine components are adjusted to match the circulating viruses as closely as possible. In addition, monitoring of antiviral susceptibility remains crucial to offer effective treatment to influenza disease.

## Introduction

Influenza viruses can cause large seasonal epidemics with high healthcare impact and severity. As influenza A viruses may cause also pandemics, influenza viruses are monitored carefully through global surveillance systems. In Europe, influenza surveillance activities are jointly coordinated by the World Health Organization Regional Office for Europe (WHO/Europe) and the European Centre for Disease Prevention and Control (ECDC). The European influenza surveillance data are presented each week on the European Respiratory Virus Surveillance Summary (ERVISS) platform [1].

Since 2022, the influenza surveillance objectives have been expanded to include also other respiratory viruses. During and after the COVID-19 pandemic, influenza surveillance has been integrated with surveillance for severe acute respiratory syndrome coronavirus 2 and respiratory syncytial virus. However, the objectives are still focused on several core aspects. Monitoring the intensity, geographical spread and temporal patterns of the influenza virus, as well as severity, risk factors for severe disease and assessment of the impact on healthcare systems is important to inform mitigation measures. In addition, monitoring changes in characteristics of circulating and emerging respiratory viruses is crucial to inform treatment, drug and vaccine development. Surveillance efforts also focus on describing the impact of disease associated with virus infections, and assessing vaccine effectiveness against influenza, COVID-19 and other respiratory virus infections where locally feasible [2].

Influenza viruses are divided in types and subtypes. Seasonal influenza surveillance collects information on influenza viruses circulating in humans, with focus on A(H1N1)pdm09 and A(H3N2) subtypes as well as B/Victoria-lineage viruses. These influenza virus types are included also in the seasonal influenza vaccines. During the 2021/22 influenza season, the circulation of influenza viruses returned [3] after very low circulation of influenza viruses during COVID-19 pandemic 2020/21 [4].

Given the recent changes in influenza virus circulation, our aim was to assess the virological characteristics of the circulating influenza viruses in the European Union/European Economic Area (EU/EEA) countries during the 2022/23 season and relate our findings to the decisions on influenza vaccine composition for 2023/24 for influenza season. Influenza virus vaccine components recommended for northern hemisphere (NH) seasons 2022/23 and 2023/24 are discussed in regard to the circulating viruses.

## Methods

### Surveillance data

We performed a descriptive analysis of the influenza virus detections and characterisations reported by National Influenza Centres (NIC) from the 30 EU/EEA countries during week 40/2022 to week 39/2023, and retrieved 5 October 2023 from The European Surveillance System (TESSy) as part of Global Influenza Surveillance and Response System (GISRS). All data originated from swabbing patients in ambulatory and inpatient populations from sentinel primary care and non-sentinel (e.g. diverse populations, including outpatients, hospitals, outbreak investigations, long-term care facilities) sources [5]. Countries reported virological influenza surveillance and strain-based antigenic characterisation data based on haemagglutination (HA) inhibition assay as well as genetic data on a weekly basis to TESSy, as described earlier [5,6]. Data are presented at the EU/EEA level. Regional differences in virus circulation within the countries may affect the virus characterisation data collected. This analysis focusses on the virological surveillance data and does not cover epidemiological aspects of the season.

### Phylogenetic analysis

Phylogenetic analysis was performed with HA sequences available within the reporting period, as described earlier [5]. In brief, a collection of reference sequences was compiled from viruses assigned at the NH WHO vaccine recommendations meeting (VCM) in February 2023 [7] and selected strains from previous years. Nextclade [8] was used to assign clade designations for the reference viruses. Sequences of 2022/23 influenza season were accessed on 5 October 2023 from GISAID EpiFlu and were aligned with the reference sequences using mafft v7. After trimming to include only the HA1 coding region, RAxML v8.2.7 was used to construct a phylogenetic tree using 10 bootstraps and a maximum likelihood search. The tree was rooted on the oldest reference sequence using treesub (https://github.com/tamuri/treesub) and PAML baseml v4.9f was used to perform ancestral reconstruction of the HA1 sequences for all internal nodes of the tree. Treesub was used to annotate the tree branches with amino acid substitutions, based on the root sequence. The resulting trees were analysed, and genetic clades were assigned by comparison with the reference sequences. For viruses without a sequence identifier, country assessment of genetic clade classification from TESSy was used instead.

### Antiviral susceptibility

Antiviral susceptibility analysis was performed as described earlier for the neuraminidase (NA) inhibitors oseltamivir and zanamivir, and for matrix-2 protein (M2) inhibitors adamantanes [9]. Susceptibility analyses were performed genotypically for the polymerase acidic (PA) protein inhibitor baloxavir marboxil. Phenotypic degrees of inhibition by NA inhibitors were determined by NA enzyme activity inhibition assay, as reported by countries, and genotypic inhibition or susceptibility was predicted by genetic markers for reduced inhibition or reduced susceptibility based on the WHO marker lists [10,11].

## Results

Influenza viruses circulated above the epidemic threshold of 10% positivity in sentinel specimens in weeks 45/2022–16/2023. In season 2022/23, 94,596 sentinel and 2,041,991 non-sentinel specimens were tested for influenza, of which 19,655 sentinel and 177,651 non-sentinel specimens tested positive in the 30 EU/EEA countries. In total, 73% of all positive specimens (n = 143,409) were type A and 27% (n = 53,897) type B viruses. The number of specimens positive for influenza in primary care sentinel and non-sentinel surveillance and proportions of positive specimens among those tested are described in Supplementary Figure S1.

Of 41,956 subtyped influenza A viruses, 36% (n = 15,242) were detected as influenza A(H1) or A(H1N1)pdm09, and 64% (n = 26,714) were influenza A(H3) or A(H3N2). Of the 53,897 reported influenza type B viruses, lineage was determined for 13% (n = 6,908) with all viruses assigned to the B/Victoria lineage (see Supplementary Figure S1).

### Antigenic and genetic characteristics of circulating influenza viruses

**Figure 1:**
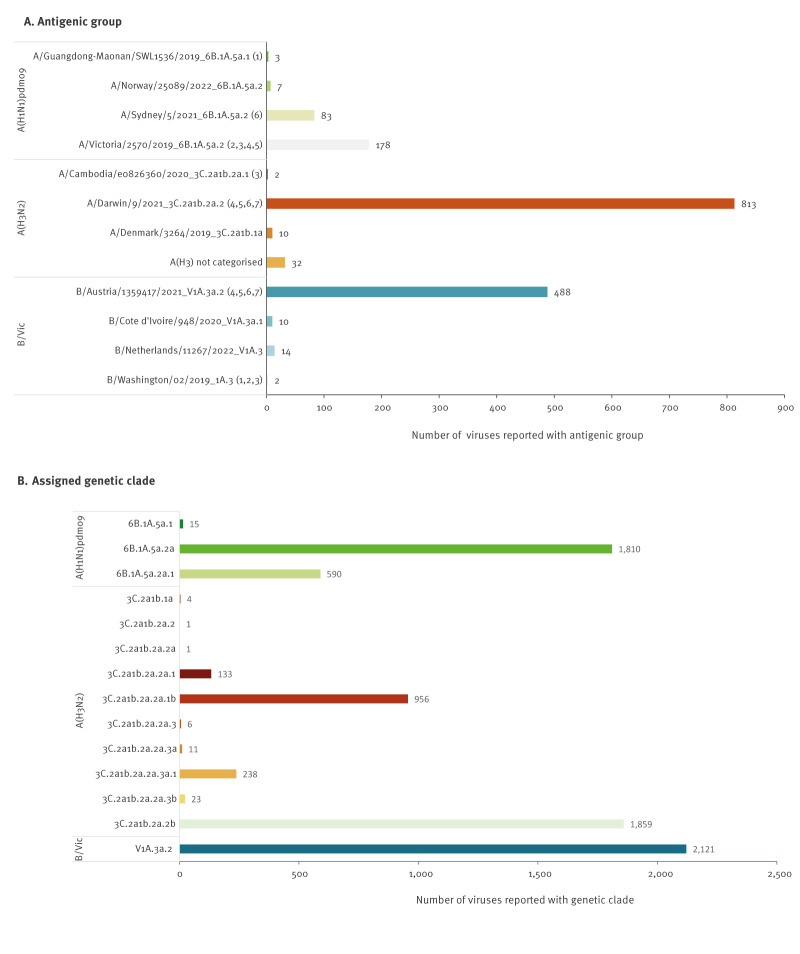
Number of viruses by antigenic group or assigned genetic clade by subtype, EU/EEA, weeks 40/2022–39/2023 (n = 1,642)

In total, 8,670 viruses underwent virus characterisation; 1,642 viruses were antigenically characterised (from eight countries) and 7,768 viruses had sequence information sufficient for clade assignment (from 15 countries, with 7,357 of those reported with genetic clade by the country) (Figure 1). Supplementary Table S1 and Supplementary Figure S2 provide the antigenic and genetic characterisation data as reported to TESSy by week of sampling and proportions by subtype. Of the 1,642 antigenically characterised viruses, 1,044 (64%) were from sentinel and 598 (36%) from non-sentinel sources. Of the genetic characterisation reports, 3,316 (43%) were from sentinel and 4,444 (57%) from non-sentinel sources. Eight viruses were reported without source.

B/Vic: B/Victoria.

The reference viruses are listed by subtype, strain name and clade for antigenic groups (A) and by assigned genetic clade based on phylogenetic analysis (B). Numbers in brackets indicate for which vaccine composition the virus was recommended by the World Health Organization: (1) 2020/21 northern hemisphere influenza season (trivalent vaccines); (2) 2021 southern hemisphere influenza season (trivalent vaccines); (3) 2021/22 northern hemisphere influenza season (trivalent vaccines); (4) 2022 southern hemisphere influenza season (trivalent vaccines); (5) 2022/23 northern hemisphere influenza season (trivalent vaccines); (6) 2023 southern hemisphere influenza season (trivalent vaccines); (7) 2023/24 northern hemisphere influenza season (trivalent vaccines).

### Influenza A(H1N1)pdm09

Of the 271 antigenically characterised A(H1N1)pdm09 viruses, 178 (65%) were reported as being like the 2022/23 NH seasons vaccine virus A/Victoria/2570/2019-component (clade 6B.1A.5a.2), 83 (31%) were A/Sydney/5/2021-like (6B.1A.5a.2a), seven (3%) were A/Norway/25089/2022-like (6B.1A.5a.2a.1) and three (1%) were A/Guangdong-Maonan/SWL1536/2019-like (6B.1A.5a.1), although geographical differences in virus circulation may affect representativeness for the EU/EEA (Figure 1). The antigenic and genetic characterisation data as reported to TESSy by week of sampling are provided in Supplementary Table S1 and proportions by subtype are provided in Supplementary Figure S2.

**Figure 2:**
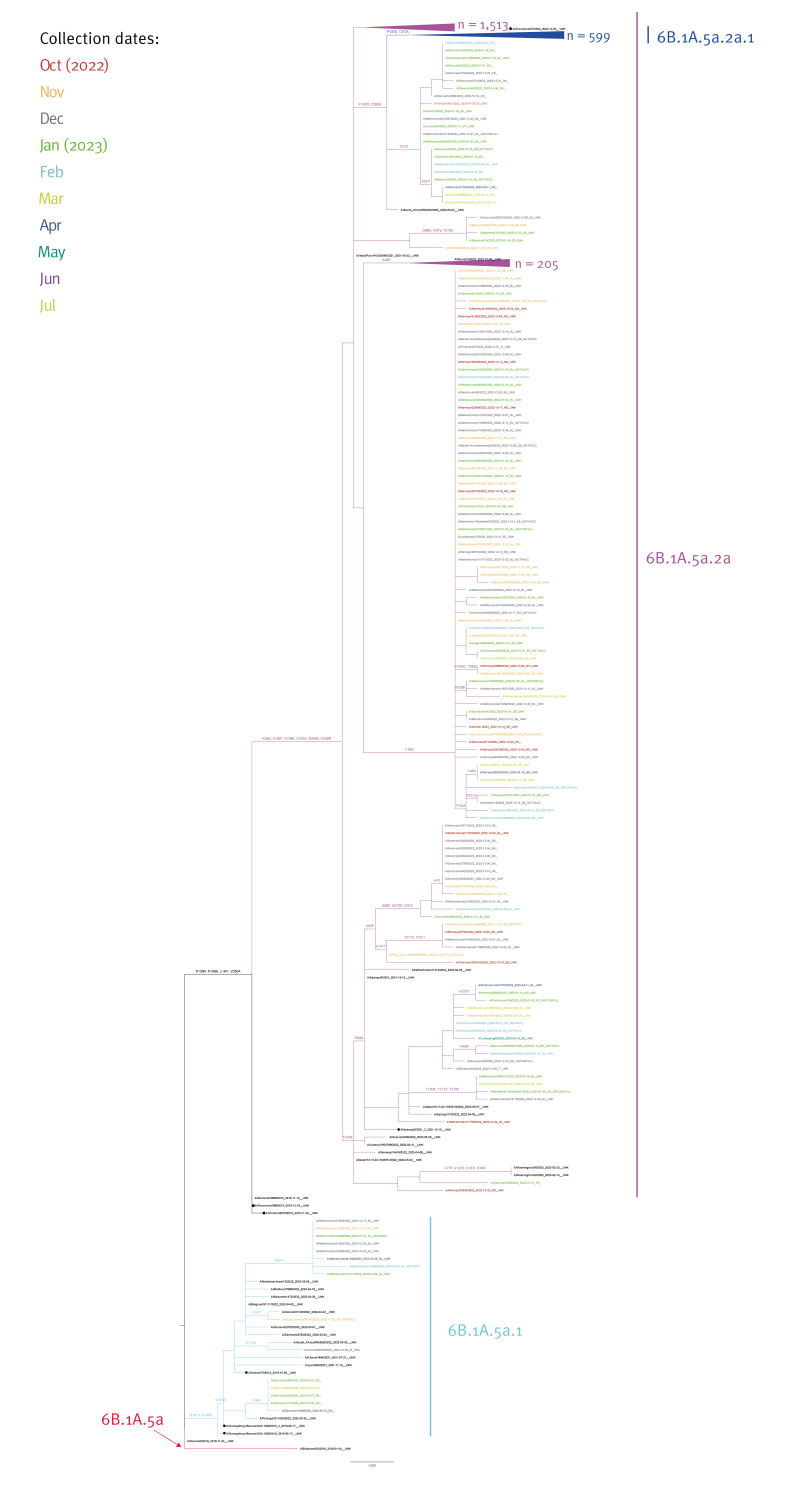
Phylogenetic comparison of influenza A(H1N1)pdm09 haemagglutinin genes, EU/EEA, weeks 40/2022–39/2023 (n = 2,466)

NOTVACC: unvaccinated; VACCINFULL: fully vaccinated; UNK: unknown vaccination status.

Phylogenetic analysis was performed with 2,466 HA gene sequences from A(H1N1)pdm09 viruses (Figure 2). Supplementary Table S2 provides the number of influenza virus haemagglutinin (HA) gene full length sequences retrieved with GISAID EpiFlu database accession number and analysed in this report, by subtype/lineage and country). Using A/Brisbane/02/2018 as the root, the results showed that all viruses belonged to the clade 6B.1A.5a with 2,451 (> 99%) belonging to 6B.1A.5a.2a, defined by the amino-acid substitutions K54Q, A186T, Q189E, E224A, R259K and K308R compared with 6B.1A.5a.2 reference virus A/Victoria/2570/2019, the A(H1N1)pdm09 component of 2022/23 NH vaccine. Fifteen (< 1%) viruses belonged to 6B.1A.5a.1 represented by reference virus A/Guangdong-Maonan/SWL1536/2019 (Figure 2).

Vaccine virus strains (solid black circles), northern hemisphere 2022/23 vaccine strains (solid black squares) and a northern hemisphere 2023/24 vaccine strain (black star) are indicated in the tree.

The number of collapsed sequences (including reference sequences) are mentioned next to the branches. Branch colouring indicates the different clades and subclades. The vaccine strains are in red, and reference strains are in black. For each virus strain, collection date and patient’s vaccination status, if known, is displayed after the virus name. All sequences reported to TESSy are coloured according to the virus collection date by month.

Within subclade 6B.1A.5a.2a, most viruses (1,513/2,466; 61%) were clustered on a branch with a high number of sub-branches but with no distinct additional amino acid substitutions; 599/2,466 (24%) viruses clustered into subclade 6B.1A.5a.2a.1, characterised by P137S, K142R, D260E and T277A which includes vaccine strain A/Wisconsin/67/2022 and 205 (8%) carried A48P similar to A/Maine/10/2022 representative virus (Figure 2).

Of the 15 viruses that belonged to 6B.1A.5a.1, eight carried V321I and five I149V compared with representative virus A/Guangdong-Maonan/SWL1536/2019. These amino-acid substitutions are not shared by any reference strain within 6B.1A.5a.1 (Figure 2).

In the first weeks of the season, the 6B.1A.5a.2a.1 clade predominated but from week 47 onward, the majority of A(H1N1)pdm09 viruses clustered with the 6B.1A.5a.2a clade in the EU/EEA (see Supplementary Figure S2). The 6B.1A.5a.1 viruses circulated at very low levels throughout the season in the EU/EEA.

### Influenza A(H3N2)

Of the 857 antigenically characterised A(H3N2) viruses, the majority (n = 813; 95%) were reported as A/Darwin/9/2021-like (3C.2a1b.2a.2), 10 (1%) were A/Denmark/3264/2019-like (3C.2a1b.1a), two (< 1%) were A/Cambodia/e0826360/2020-like and 32 viruses (4%) were not attributed to any of the reporting categories (Figure 1). The antigenic and genetic characterisation data as reported to TESSy by week of sampling are provided in Supplementary Table S1 and Supplementary Figure S2. Of the 32 viruses without reporting category, eleven were genetically similar to A/Darwin/9/2021 and a subset of these viruses were sent to the WHO Collaborating Centre in London, United Kingdom for further characterisation. Questions on the remaining viruses were clarified with the reporting country, which confirmed that viruses did not deviate antigenically from the vaccine virus.

**Figure 3:**
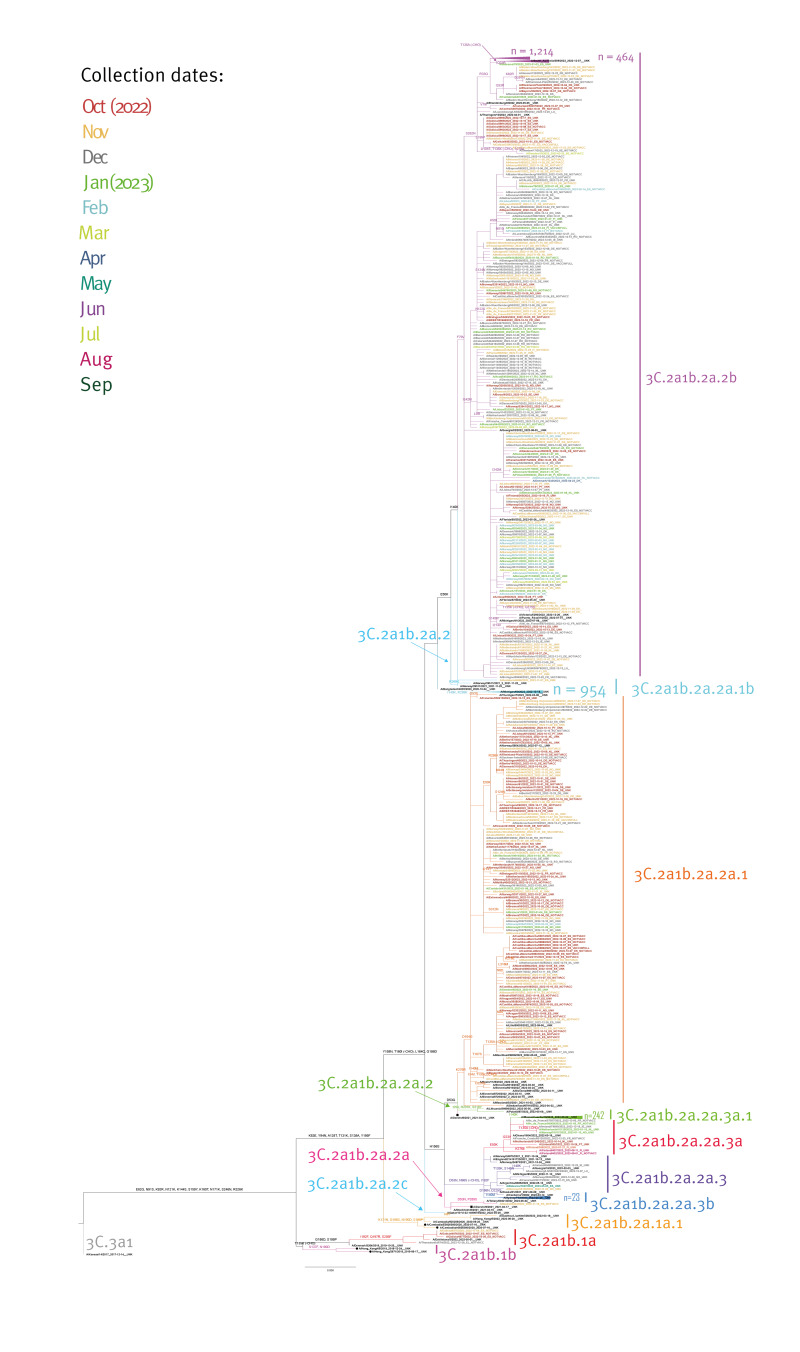
Phylogenetic comparison of influenza A(H3N2) haemagglutinin genes, EU/EEA, weeks 40/2022–39/2023 (n = 3,240)

NOTVACC: unvaccinated; VACCINFULL: fully vaccinated; UNK: unknown vaccination status.

Phylogenetic analysis conducted with 3,240 HA gene sequences from A(H3N2) viruses (provided in Supplementary Table S2), where A/Kansas/14/2017 is the root, showed that nearly all belonged to the clade 3C.2a1b.2a.2 (> 99%; n = 3,236) defined by the amino acid substitutions Y159N, T160I (resulting in the loss of a glycosylation site), L164Q and G186D. Four viruses (< 1%) belonged to clade 3C.2a1b.1a (Figure 3).

Vaccine virus strains (solid black circles) and northern hemisphere 2022/23 vaccine strains (solid black squares) are indicated in the tree.

The number of collapsed sequences (including reference sequences) are mentioned next to the branches. Branch colouring indicates the different clades and subclades. The vaccine strains are in red, and reference strains are in black. For each virus strain, collection date and patient’s vaccination status, if known, is displayed after the virus name. All sequences reported to TESSy are coloured according to the virus collection date by month.

The majority of viruses (58%; n = 1,865) belonging to 3C.2a1b.2a.2 were assigned into clade 3C.2a1b.2a.2b, characterised by substitutions E50K, F79V and I140K and represented by A/Thuringen/10/2022. This clade was predominant for the majority of the season within the A(H3N2) viruses in the EU/EEA. Supplementary Figure S2 shows the proportion of viruses by assigned clade by subtype and week. The remaining (42%; n = 1,370) fell into 3C.2a1b.2a.2a, characterised by H156S and represented by A/Darwin/9/2021. All except one virus within 3C.2a1b.2a.2a could be further characterised into the following sub-clades: 2a.1b (n = 954), 2a.3a.1 (n = 242), 2a.1 (n = 133), 2a.3b (n = 23), 2a.3a (n = 11) and 2a.3 (n = 6) (Figure 3).

Most viruses within 3C.2a1b.2a.2b (64%; n = 1,214) carried R33Q and no reference strain was present on this branch. A substantial number of 3C.2a1b.2a.2b (n = 464) carried the amino acid substitution T135A (and lost R33Q in a reversion), which leads to a loss of glycosylation site, similar to A/South Australia/389/2022 (Figure 3).

### Influenza B

Among 514 antigenically characterised influenza B viruses, all were from Victoria lineage, and the majority (95%; n = 488;) were similar to the vaccine virus component for the 2022/23 season B/Austria/13594177/2021 (Figure 1). The antigenic and genetic characterisation data as reported to TESSy by week of sampling are provided in Supplementary Table S1 and proportions by subtype are provided in Supplementary Figure S2.

Fourteen viruses (3%) were B/Netherlands/11267/2022-like, 10 (2%) were B/Cote d’Ivoire/948/2020-like and two (< 1%) were B/Washington/02/2019-like.

**Figure 4:**
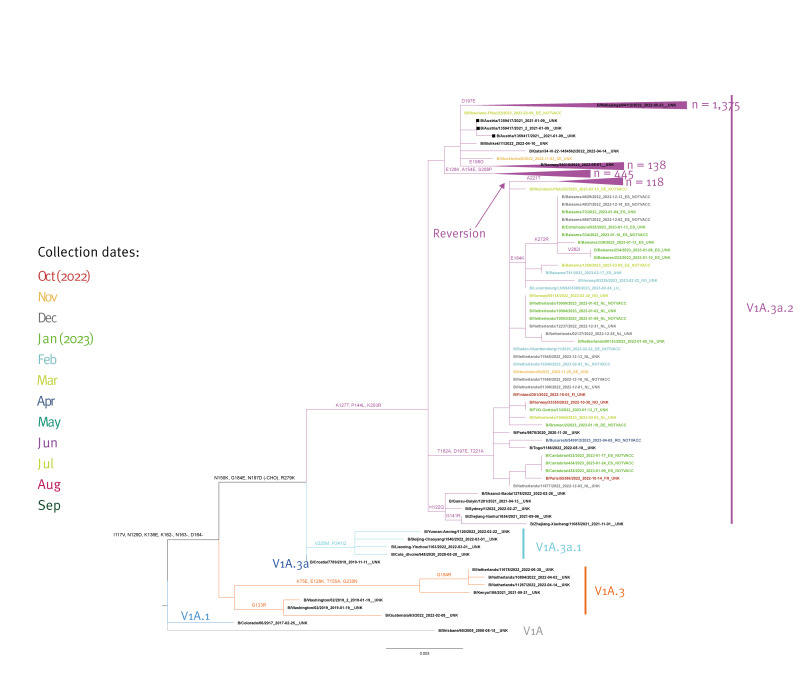
Phylogenetic comparison of influenza B/Victoria-lineage haemagglutinin gene, EU/EEA, weeks 40/2022–39/2023 (n = 2,116)

NOTVACC: unvaccinated; VACCINFULL: fully vaccinated; UNK: unknown vaccination status.

Phylogenetic analysis performed with 2,116 HA gene sequences from B/Victoria viruses, where B/Brisbane/60/2008 is the root, showed that all viruses fell into genetic clade V1A.3 subgroup 3a.2. with characteristic amino acid substitutions A127T, P144L and K203R (Figure 4).

Northern hemisphere 2022/23 vaccine strains (solid black squares) are indicated in the tree.

The number of collapsed sequences (including reference sequences) are mentioned next to the branches. Branch colouring indicates the different clades and subclades. The vaccine strains are in red, and reference strains are in black. For each virus strain, collection date and patient’s vaccination status, if known, is displayed after the virus name. All sequences reported to TESSy are coloured according to the virus collection date by month.

Diversity within V1A.3a.2 was shown as four distinct branches where the major one (1,375/2,116 viruses; 65%) was defined by the D197E substitution similar to B/Mahajanga/04112/2022. Within this branch, 407 (30%) viruses additionally carried E183K and included the reference B/Catalonia/2279261NS/2023. The second most populated branch was defined by E128K, A154E and S208P and contained 445/2,116 (20%) viruses but no current reference strain. The third branch included 156 (7%) viruses and was defined by T182A, D197E and T221A similar to B/Paris/9878/2020, however most (n = 118; 76%) clustered into a sub-branch with a A221T reversion but no reference virus. The fourth branch had the amino acid substitution E198G (n = 138; 6.5%) along with reference virus B/Norway/29315/2022. (Figure 4)

### Antiviral susceptibility

Since the beginning of the season, 6,768 viruses were assessed for susceptibility to neuraminidase inhibitors oseltamivir and/or zanamivir, and/or PA inhibitor baloxavir marboxil. The reduced inhibition/susceptibility following antiviral susceptibility testing reported to TESSy by subtype and drug tested are provided in Supplementary Table S3. In total, 10 viruses with reduced or highly reduced inhibition were detected. Nine viruses exhibited reduced or highly reduced inhibition by oseltamivir (eight A(H1)pdm09, one A(H3)); one of the A(H1)pdm09 viruses and one additional B/Victoria virus showed reduced inhibition by zanamivir. The antiviral susceptibility by subtype and list of viruses reported with reduced inhibition with interpretation defining amino acid substitutions are provided in Supplementary Table S3 and Supplementary Table S4, respectively. No virus showed genotypically reduced susceptibility to baloxavir marboxil. Additionally, 2,019 influenza A viruses were assessed for susceptibility to adamantanes, all of which showed genotypically highly reduced inhibition.

## Discussion

After very low circulation of influenza viruses in Europe during the season of 2020/21 [3] and an A(H3N2)-dominated season in 2021/22 [12], the influenza season of 2022/23 was characterised by co-circulation of different influenza (sub)types and a succession of influenza A dominance followed by a dominance of influenza B. The first peak of the season was primarily caused by influenza A viruses in week 51/2022. The numbers decreased thereafter and an increase, notably of B viruses, was observed from week 03/2023 onwards. No detection of B/Yamagata viruses was reported. Thus, the season of 2022/23 was characterised by a rather unusual bi-phasic pattern with two peaks observed during the season, and even abrupt decreases in influenza transmission below epidemic thresholds were detected in some countries [12,13]. 

Predominant clades were 6B.1A.5a.2a for A(H1N1)pdm09 viruses, 3C.2a1b.2a.2b for A(H3N2) and V1A.3a.2 for the B/Victoria-lineage, however, there were differences in circulation across countries. Human serology studies presented at the NH VCM in February 2023 for A(H1N1)pdm09 viruses demonstrated notable reduced post-vaccination geometric mean titres against multiple recently circulating clade 5a.2a and 5a.2a.1 A(H1N1)pdm09 viruses when compared with titres against cell culture-propagated A/Wisconsin/588/2019 or egg-propagated A/Victoria/2570/2019-like vaccine viruses [7]. Given inefficient recognition of the circulating A(H1N1)pdm09 clade 6B.1A.5a.2 vaccine viruses, the vaccine A(H1N1)pdm09 component was updated to match the recently emerged subclades 6B.1A.5a.2a and 6B.1A.5a.2a.1 more closely [7]. Subclade 6B.1A.5a.2a carries additional HA1 amino acid substitutions K54Q, A186T, Q189E, E224A, R259K and K308R, of which some are located in antigenic site Sb [7]. Subclade 6B.1A.5a.2a.1 viruses have acquired additional amino acid substitutions P137S, K142R, D260E and T277A and are represented by the new vaccine viruses A/Wisconsin/67/2022 and A/Victoria/4897/2022. In our dataset, these viruses represented one fourth of the A(H1N1)pdm09 viruses. Antigenically, the genetically diversified A(H3N2) and B/Victoria-lineage viruses are efficiently covered by the vaccine components for NH 2023/24 season [7].

Interim results for vaccine effectiveness (VE), produced by the Vaccine Effectiveness, Burden and Impact Studies (VEBIS) network for the 2022/23 season, indicated a high VE against influenza B (51%; 95% CI: 1 to 79) in an EU primary care study, with estimates among children at 88% (95% CI: 47 to 97), and lower for A(H1N1)pdm09 (ranging from 28% (95% CI: 0 to 50) to 46% (95% CI: 26 to 60)) or A(H3N2) (ranging from 2% (95% CI: −53 to 37) to 44% (95% CI: 32 to 54)) when all ages and study sites were merged [14]. In Wisconsin, United States, the vaccine effectiveness estimates for this season, where circulation of both A(H1N1)pdm09 (25%) and A(H3N2) (75%) were detected, was 54% against medically-attended outpatient acute respiratory illness associated with laboratory-confirmed influenza A among patients aged 6 months–64 years, and 71% against symptomatic laboratory-confirmed influenza A virus infection [15]. In a Canadian study for the current 2022/23 season, vaccine effectiveness for A(H3N2) viruses was found to be 54% (95% CI: 38 to 66) overall with potential variation based on HA gene amino acid position 135 genetic diversity [16]. It is important to note that national or sub-national level VE results are dependent on the circulating strains in that region and, thus, there might be large differences between countries. These data show that 16% (506/3,240) of the sequenced A(H3N2) viruses carry the T135A mutation in the HA gene and an additional 11 carry T135K; these substitutions, which cause a loss of a glycosylation site, could lead to lower vaccine effectiveness.

All but 10 of 6,768 viruses tested during 2022/23 season in our dataset remained sensitive to NA and PA inhibitors (oseltamivir, zanamivir and baloxavir marboxil) and all tested influenza A viruses remained resistant to M2 inhibitors. While vaccination against influenza remains the best means to protect against severe disease, NA and PA inhibitors should be considered for clinical management of influenza, especially in high-risk and elderly patients (aged ≥ 65 years) regardless of their vaccination status.

Since March 2020, no B/Yamagata-lineage viruses have been detected globally [17,18]. At the September 2023 VCM, the WHO influenza vaccine composition advisory committee stated that the inclusion of B/Yamagata-lineage antigens in influenza vaccines is no longer warranted, and it should be excluded in the upcoming composition [19,20]. All necessary precautions as per standard operation procedures need to be taken when sending or handling potentially infectious influenza B/Yamagata virus (e.g. for EQA, research or validation purposes) to make sure not to release infectious B/Yamagata into the population [21]. In Europe, efforts to determine the lineage of B viruses need to be strengthened, as only 13% of detected B viruses (and 31% of the sentinel B virus detections) were lineage-determined. The current ECDC/WHO surveillance guidance recommends sequencing all sentinel specimens if resources allow [22]. In addition, reference laboratories should also be alerted about the possibility of detecting live attenuated influenza vaccine B/Yamagata-lineage (or other vaccine component) viruses to perform whole genome sequencing and to report those separately with comments to TESSy. 

When comparing the level of virus characterisations to the average of 2016/17 to 2019/20 seasons, there was a twofold increase of genetic reports. Antigenic reports were approximately at the same level as the average of the pre-COVID-19 seasons. In total, 16% of detected sentinel surveillance viruses were characterised genetically, and 5% antigenically [22]. However, surveillance systems in many countries are still impacted by the COVID-19 pandemic. Although, an increase in sequencing efforts has been observed, several factors affect the capacity for sequencing. For example, laboratories apply other criteria than surveillance source for selection of specimens for further characterisation, e.g. PCR Cq value below 25, to increase the success rate in sequencing. For antigenic characterisation, the recommendation is to select a subset of genotyped viruses for antigenic characterisation [22] and every twentieth specimen has been successfully analysed. Importantly, the number of antigenic characterisation reports increased in 2022/23 again to levels observed in 2018/19 (n = 1,643 and 1,699 reports, respectively) after a marked decrease in 2019/20 (n = 1,022 reports). The increase was observed although the NIC terms of reference do not include antigenic characterisation [23]. Furthermore, more countries are again performing antigenic characterisation since 2019/20.

The reasons for an almost twofold increase in sequencing volume compared to the pre-COVID-19 time is likely a result of sequencing capacity increases in the EU/EEA that have been fostered during the COVID-19 pandemic. These new sequencing capacities and capabilities improve the earlier surveillance systems if the sampling for genetic characterisation is done in a representative way. From the pool of genetically characterised viruses, a specific subset of viruses of interest can then be selected for antigenic characterisation and phenotypic antiviral susceptibility testing. This is more efficacious and cost-effective than the earlier antigenic characterisation-first approach.

Certain limitations apply to this study. Firstly, only half of the EU/EEA countries reported influenza virus characterisation data, and to a varying extent. Secondly, national patterns for influenza season characteristics differ partly from the overall EU/EEA season in terms of virus dominance, timing and shape of the epidemic curve. Thirdly, the specimen sources (sentinel general practitioners, hospital, intensive care units, outbreak investigations) and selection processes for the viruses that undergo characterisation might vary from country to country. Therefore, the presented data may not be fully representative of the circulating strains in the region, even if surveillance efforts aim to cover the whole populations. Certain selection bias for sampling has likely occurred and selection for virus characterisation may be biased towards vaccine escape mutants and from more severe patients.

## Conclusion

Influenza virus surveillance data provide an important mechanism for monitoring the evolution of influenza viruses over the season and provide crucial data for the vaccine composition decision which led to the change of the A(H1N1)pdm09 component for the NH 2023/24 influenza season. Identifying genetic and antigenic mismatches between the circulating and vaccine strains also gives the opportunity to anticipate the vaccine effectiveness results of the current season. Laboratories and national public health institutes should continue their efforts to collect data on influenza and on virus characterisation and to provide representative specimens to WHO collaborating centres to support the biannual decision processes for recommendations for the influenza vaccine compositions. Efforts in standardising the selection of specimens for sequencing and antigenic characterisation across the countries could improve results and therefore future efforts should be made to improve randomness or representativeness of the sequences to improve quality of virological influenza surveillance data.

## Members of the European Reference Laboratory Network for Human Influenza (ERLI-Net)

Denmark: Amanda Bolt Botnen and Ramona Trebbien, Statens Serum Institut, Copenhagen.

Finland: Niina Ikonen and Erika Lindh, Finnish Institute for Health and Welfare (THL), Helsinki.

France: Vincent Enouf, National Reference Center of Respiratory Viruses - Institut Pasteur, Paris, and Laurence Josset, National Reference Center of Respiratory Viruses - Hospices Civils de Lyon, Lyon.

Germany: Ralf Dürrwald and Marianne Wedde, Robert Koch Institute, Berlin.

Greece: Maria Exindari, National influenza Centre for N. Greece, Thessaloniki and Mary Emmanouil, National Reference Laboratory for S. Greece, Hellenic Pasteur Institute, Athens.

Ireland: Elaine Brabazon, Health Service Executive, Health Protection Surveillance Centre, Dublin, and Charlene Bennett, National Virus Reference Laboratory, University College Dublin.

Italy: Simona Puzelli and Marzia Facchini, Institute of Health (Istituto Superiore di Sanità), Rome.

The Netherlands: Ron Fouchier, Erasmus University Medical Center, Rotterdam, and Adam Meijer, Dutch National Institute for Public Health and the Environment (RIVM), Bilthoven.

Norway: Andreas Rohringer and Karoline Bragstad, Norwegian Institute of Public Health, Oslo.

Portugal: Raquel Guiomar, National Reference Laboratory for Influenza and Other Respiratory Viruses, Infectious Diseases Department, and Ana Paula Rodrigues, Department of Epidemiology, National Institute of Health Doctor Ricardo Jorge.

Romania: Mihaela Lazar, ‘Cantacuzino’ National Medical-Military Research-Development Institute, Rodica Popescu, National Institute of Public Health Romania.

Slovenia: Katarina Prosenc, National Laboratory for Health, Environment and Food, Ljubljana and Nataša Berginc, National Laboratory for Health, Environment and Food, Ljubljana.

Spain: Francisco Pozo and Inmaculada Casas, National Centre for Microbiology, Institute of Health Carlos III, Madrid. Consortium for Biomedical Research in Epidemiology and Public Health (CIBERESP).

Sweden: Tove Samuelsson-Hagey and Neus Latorre-Margalef, Public Health Agency of Sweden, Stockholm.

ECDC: Cornelia Adlhoch.

WHO CC for reference and research on influenza, Crick Worldwide Influenza Centre, The Francis Crick Institute, London, United Kingdom: Monica Galiano and Nicola Lewis.

## Supplementary Data

316000 Supplement
