# Supplementary material for: Co-circulation of seasonal influenza A(H1N1)pdm09, A(H3N2) and B/Victoria lineage viruses with further genetic diversification, EU/EEA, 2022/23 influenza season
Source: Euro Surveill. 2024 Sep 26;29(39):2400020. doi: 10.2807/1560-7917.ES.2024.29.39.2400020 (PMC11484342; doi:10.2807/1560-7917.ES.2024.29.39.2400020)
Supplement: Supplement [file 24-00020_BROBERG_Supplement.pdf]

This supplementary material is hosted by *Eurosurveillance* as supporting information alongside the article *Co-circulation of seasonal influenza A(H1N1)pdm09, A(H3N2) and B/Victoria-lineage viruses with further genetic diversification, EU/EEA, 2022/23 influenza season*, on behalf of the authors, who remain responsible for the accuracy and appropriateness of the content. The same standards for ethics, copyright, attributions and permissions as for the article apply. Supplements are not edited by *Eurosurveillance* and the journal is not responsible for the maintenance of any links or email addresses provided therein.

Supplemental Table ST1. Antigenic and genetic characterisation data as reported to TESSy by week of sampling, EU/EEA, weeks 40/2022-39/2023. Blue indicates antigenic, green indicates genetic characterisations based on TESSy reported sequences and/or genetic clade data.

		ANTIGENIC													GENETIC														
		A(H1)pdm09				A(H3)				B/Victoria					A(H1)pdm09			A(H3)										B/Victoria	
YEAR	WEEK	AH1/Guangdong-Maonan/SWL1536/2019_6B.1A.5a.1 (1)	AH1/Sydney/5/2021_6B.1A.5a.2 (6)	AH1/Victoria/2570/2019_6B.1A.5a.2 (2,3,4,5)	AH1/Norway/25089/2022_6B.1A.5a.2a.1	AH3/Cambodia/e0826360/2020_3C.2a1b.2a.1a (3)	AH3/Darwin/9/2021_3C.2a1b.2a.2 (4,5,6)	AH3/Denmark/3264/2019_3C.2a1b.1a	AH3NOCAT	BVic/Austria/1359417/2021_V1A.3a.2 (4,5,6)	BVic/Cote d'Ivoire/948/2020_V1A.3a.2	BVic/Washington/02/2019_V1A.3	BVic/Netherlands/11267/2022_V1A.3	TOTAL ANTIGENIC	6B.1A.5a.1 (1)	6B.1A.5a.2a (6)*	6B.1A.5a.2a.1	3C.2a1b.1a (2)**	3C.2a1b.2a.2 (4,5,6)3	3C.2a1b.2a.2a (4)***	3C.2a1b.2a.2a.1	3C.2a1b.2a.2a.1b	3C.2a1b.2a.2a.3	3C.2a1b.2a.2a.3a	3C.2a1b.2a.2a.3a.1	3C.2a1b.2a.2a.3b	3C.2a1b.2a.2b	V1A.3a.2 (4,5,6)	TOTAL GENETIC
2022	40	1					26			1				28		8	23	2			24	14			7	1	70	3	152
	41			3			28			3				34		6	23				16	21		1	3	3	53	8	134
	42			3			38							41		6	25				12	15			10	1	74	2	145
	43			1			39		1	1				42		12	27				5	14		1	9		54	6	128
	44			1			36		4					41		12	23	1			8	30	1		5		96	10	186
	45			2			64		1	2				69		27	29		1		17	49			13	2	117	3	258
	46		2	4			72		1	3				82	1	20	24			1	2	41			10	1	112	9	221
	47	1		6			92		7	3				109	1	49	27				10	51			16		134	18	306
	48			15			85		6	3				109		68	27				11	60			12	1	170	26	375
	49		1	15			59		9					84		71	52				8	75	2	1	13	1	156	36	415
	50			18			69			2				89	1	80	42				4	92	1		27	1	213	56	517
	51		1	16			64		1	1				83	1	95	32				6	101	1	2	17	3	198	47	503
	52		2	5			39		1	9				56	2	67	23	1			3	51			10	1	107	73	338
2023	1	1	21	8			30	3	1	10			3	77	2	116	20				2	45		1	13		72	84	355
	2		12	6	1		20			11			2	52		105	22				2	42		2	11	1	37	89	311
	3		10	3		1	15	1		14	8	1		53		105	16					32			7	1	31	92	284
	4		9	5	4	1	12	4		33			1	69	2	91	16				1	32			4	3	26	79	254
	5		4	8			7			35				54	1	134	8				1	43			3	2	33	179	404
	6		2	9			2			35				48		87	14					19			1		27	122	270
	7		6	7	1		6			43			4	68	1	106	16					24			2		23	138	310
	8		2	10				1		51				64		90	12				1	28			3		11	147	292
	9		1	8			2			34			3	48		69	9					11					13	140	242
	10		3	5	1		2	1		32				44		67	5					20			1		12	128	233
	11		6	5			1			28				40	1	56	9					6			1		5	130	208
	12			4						31				35		26	7					4			1		3	99	140
	13			1			1			31		1		34		17	4					9			1		4	70	105
	14									22				22	1	24	4					7			1		1	44	82
	15			2						9				11	1	37	1					10					1	41	91
	16			1			3			15				19		31	3					2					3	50	89
	17		1	4						11			1	17		21	5					1					2	45	74
	18									6				6		8	1					2	1		1		1	35	49
	19									4	1			5		28	4					1			2			35	70
	20			2						3				5		14	3					1				1		17	36
	21									1				1		11	5					2			1			24	43
	22										1			1		8												13	21
	23									1				1		1	1											11	13
	24			1			1							2		3	3											5	11
	25													0		2	2											1	5
	26													0		5	1												6
	27													0		4	1					1			1			1	8
	28													0		3	2								2			1	8
	29													0		2	4								1				7
	30													0		1	1							1	7			1	11
	31													0		4	2							1	5			1	13
	32													0		6	4							1	4			1	16
	33													0		1	7								4				12
	34													0		1	1								6			1	9
	35													0											1				1
	36													0		3									1				4
	37													0		2									1				3
	38													0															0
	39													0															0
	Total	3	83	178	7	2	813	10	32	488	10	2	14	1642	15	1810	590	4	1	1	133	956	6	11	238	23	1859	2121	7768

1 WHO recommended vaccine viruses for the 2020-21 Northern Hemisphere influenza season (Trivalent vaccines)
2 WHO recommended vaccine viruses for the 2021 Southern Hemisphere influenza season (Trivalent vaccines)
3 WHO recommended vaccine viruses for the 2021-22 Northern Hemisphere influenza season (Trivalent vaccines)
4 WHO recommended vaccine viruses for the 2022 Southern Hemisphere influenza season (Trivalent vaccines)
5 WHO recommended vaccine viruses for the 2022-23 Northern Hemisphere influenza season (Trivalent vaccines)
*6 WHO recommended vaccine viruses for the 2023 Southern Hemisphere influenza season (Trivalent vaccines) *6B.1A.5a.2 was included in 2,3,4,5 (A(H1N1)pdm09 component A/Victoria/2570/2019), **3C.2a1b.1b was included in 1,2 (A(H3N2) component A/Hong Kong/45/2019) and ***3C.2a1b.2a.1a was included in 3 (A(H3N2) component A/Cambodia/e0826360/2020)*

Supplemental table ST2.

Number of influenza virus haemagglutinin (HA) gene full length sequences retrieved with GISAID EpiFlu database accession number and analysed in this report, by subtype/lineage and country, EU/EEA, weeks 40/2022 to 39/2023.

Country/HA sequences	A(H1N1)pdm09	A(H3N2)	B/Victoria
Austria	13	5	
Denmark	341	187	369
Finland	32	60	7
France	47	231	15
Germany	79	575	220
Greece		15	
Ireland	69	86	
Italy	14	23	7
Luxembourg	21	190	161
Netherlands	581	322	439
Norway	494	292	346
Portugal	49	197	34
Romania	146	91	17
Slovenia	47	51	2
Spain	431	826	405
Sweden	101	88	94
**Total number of HA sequences**	**2466**	**3240**	**2116**

Supplemental table ST3. Influenza subtypes and lineages with and without reduced inhibition/susceptibility following antiviral susceptibility testing reported to TESSy by drug tested, EU/EEA, weeks 40/2022 to 39/2023. HRI: Highly reduced inhibition; RI: Reduced inhibition; NI: Normal inhibition; RS: Reduced susceptibility; NS: Normal susceptibility; INP: Interpretation not possible because full NA or PA segment was not sequenced; prefix 'AA': Amino acid, refers to genotypic testing result. For isolates which were tested both phenotypically and genotypically, only the phenotypical result is reported in this table.

Oseltamivir	HRI n (%)	NI n (%)	AAHRI n (%)	AARI n (%)	AANI n (%)	AAINP n(%)	Total
A(H1)pdm09	2 (0.1%)	249 (10.7%)	5 (0.2%)	1 (0%)	2080 (89%)	0 (0%)	2337
A(H3)	1 (0%)	399 (19%)	0 (0%)	0 (0%)	1695 (80.9%)	0 (0%)	2095
B/Vic	0 (0%)	370 (19.4%)	0 (0%)	0 (0%)	1540 (80.6%)	0 (0%)	1910
Total	3 (0%)	1018 (16.1%)	5 (0.1%)	1 (0%)	5315 (83.8%)	0 (0%)	6342
**Zanamivir**	**HRI n (%)**	**NI n (%)**	**AAHRI n (%)**	**AARI n (%)**	**AANI n (%)**	**AAINP n(%)**	**Total**
A(H1)pdm09	0 (0%)	229 (9.8%)	0 (0%)	1 (0%)	2105 (90.1%)	0 (0%)	2335
A(H3)	0 (0%)	387 (18.6%)	0 (0%)	0 (0%)	1696 (81.4%)	0 (0%)	2083
B/Vic	0 (0%)	359 (18.9%)	0 (0%)	1 (0.1%)	1544 (81.1%)	0 (0%)	1904
Total	0 (0%)	975 (15.4%)	0 (0%)	2 (0%)	5345 (84.5%)	0 (0%)	6322
**M2-Blocker**	**HRI n (%)**	**NI n (%)**	**AAHRI n (%)**	**AARI n (%)**	**AANI n (%)**	**AAINP n(%)**	**Total**
A(H1)pdm09	0 (0%)	0 (0%)	865 (100%)	0.00	0 (0%)	0 (0%)	865
A(H3)	0 (0%)	0 (0%)	1154 (100%)	0.00	0 (0%)	0 (0%)	1154
B/Vic*	-	-	-	-	-	-	-
Total	0 (0%)	0 (0%)	(100%)	0.00	0 (0%)	0 (0%)	2019
**Baloxavir marboxil**	**-**	**-**	**-**	**AARS n (%)**	**AANS n (%)**	**AAINP n (%)**	**Total**
A(H1)pdm09	-	-	-	0 (0%)	1231 (96.5%)	44 (3.5%)	1275
A(H3)	-	-	-	0 (0%)	1982 (99%)	21 (1%)	2003
B/Vic	-	-	-	0 (0%)	1242 (93%)	94 (7%)	1336
Total	-	-	-	0 (0%)	4455 (96.6%)	159 (3.4%)	4614

*: M2 blockers are not relevant for influenza B viruses. Detected HRI for M2 blockers were genotypic predictions based on presence of mutations M2:L26F, M2:V27I, M2:V27A and/or M2:S31N.

Supplemental table ST4. List of viruses reported with reduced inhibition or susceptibility by antiviral, subtypes and lineages, phenotypic or genotypic testing as well as corresponding GISAID id and interpretation defining mutation. TESSy, weeks 40/2022 to 39/2023, EU/EEA. AST: Antiviral susceptibility testing; HRI: Highly reduced inhibition; RI: Reduced inhibition; prefix 'AA': Amino acid, refers to genotypic testing result. N/A: Not available.

Oseltamivir					
**GISAID ID**	**Virus**	**AST**	**Interpretation**	**NAISD**	**NAAAMutations**
A/Netherlands/10294/2023	A(H1)pdm09	Phenotypic	HRI	EPI2660412	D199G (24%), H275Y (75%)
A/Schweiz/1/2023	A(H1)pdm09	Phenotypic	HRI	EPI2459759	H275Y
A/Arad/547046/2023	A(H3)	Phenotypic	HRI	N/A	N/A
A/Athens.GR/72/2023	A(H1)pdm09	Genotypic	AAHRI	EPI2689324	H275Y
A/Athens.GR/73/2023	A(H1)pdm09	Genotypic	AAHRI	EPI2689325	H275Y
A/Norway/05139/2023	A(H1)pdm09	Genotypic	AAHRI	EPI2529589	H275Y
A/PaisVasco/4350/2022	A(H1)pdm09	Genotypic	AAHRI	EPI2273970	H275Y
A/Arges/545719/2023	A(H1)pdm09	Genotypic	AAHRI	EPI2547045	H275Y
A/CastillaLaMancha/934/2023	A(H1)pdm09	Genotypic	AARI	EPI2520166	I223R
**Zanamivir**					
**GISAID ID**	**Virus**	**AST**	**Interpretation**	**NAISD**	**NAAAMutations**
A/CastillaLaMancha/934/2023	A(H1)pdm09	Genotypic	AARI	EPI2520166	I223R
B/Luxembourg/LNS8679822/2023	B/Vic	Genotypic	AARI	EPI2683836	G407S

Supplemental figure SF1. Number of specimens positive for influenza in primary care (A) sentinel and (B) non-sentinel surveillance and proportions of positive specimens among those tested, EU/EEA, week 40/2022 – week 39/2023. Viruses reported as A(H1)pdm09 or A(H3) have not been subtyped for neuraminidase (NA) gene, while those reported as A(H1N1)pdm09 or A(H3N2) have been subtyped also for NA gene.

A.

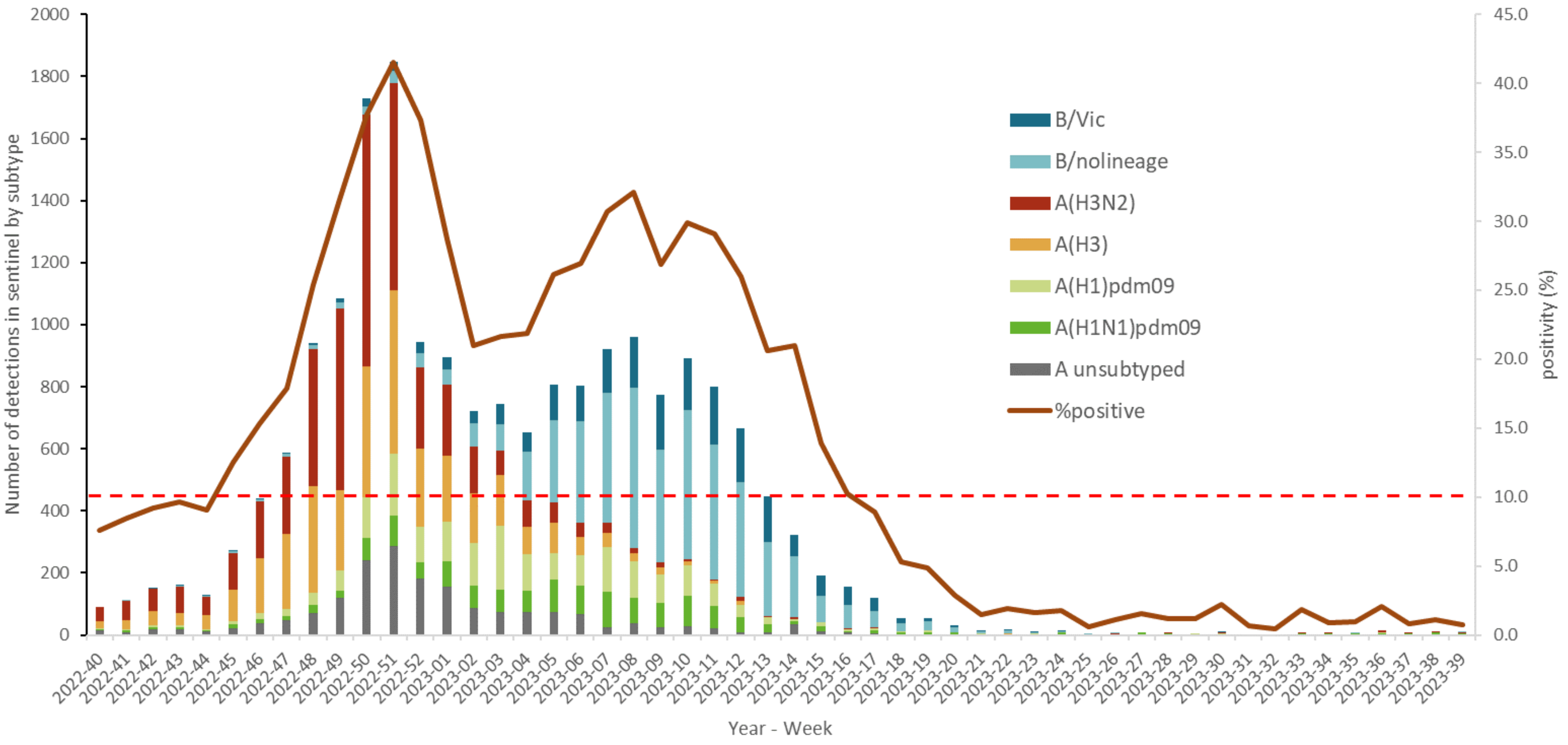

Broberg et al.

B.

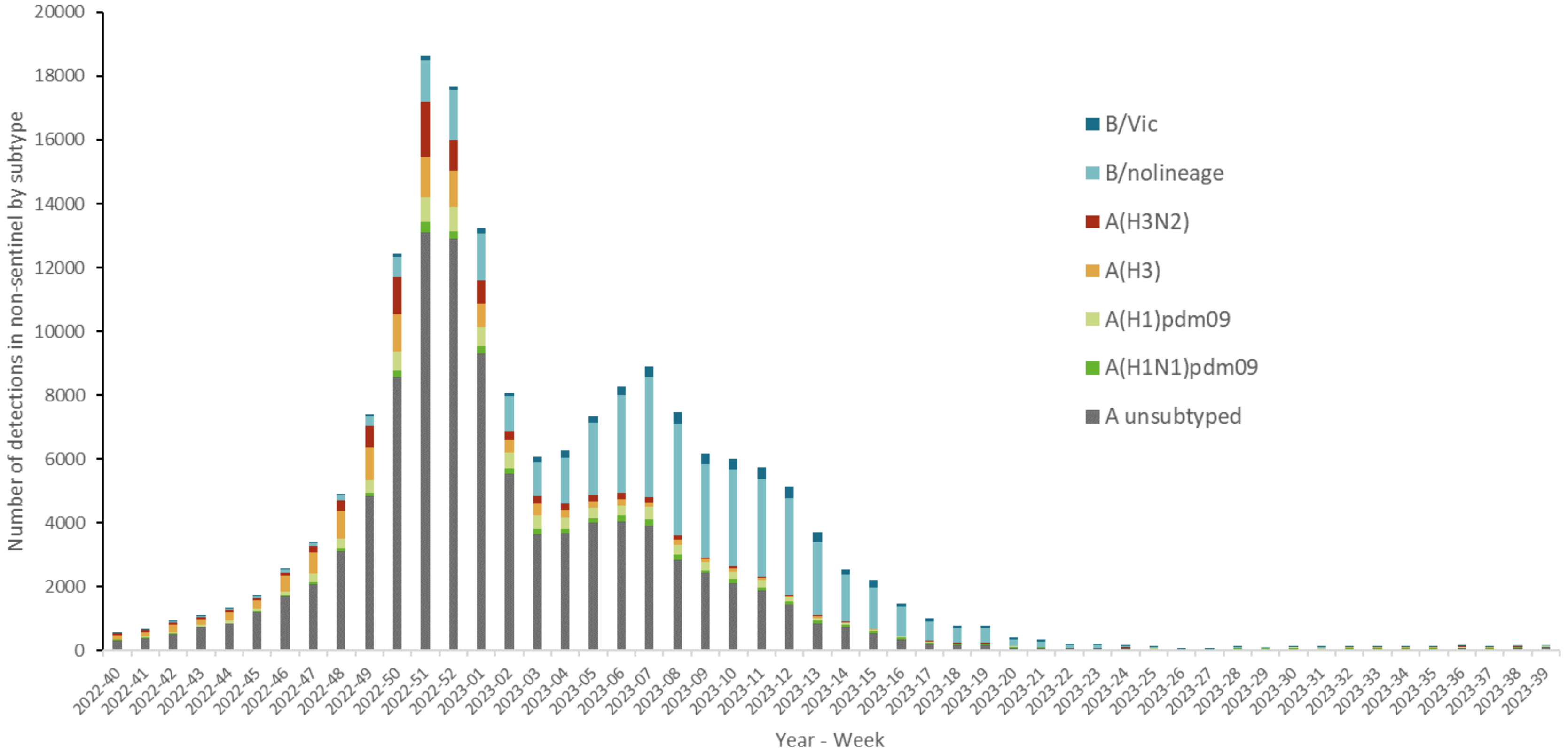

Broberg et al.

Supplemental figure SF2. Proportion (number of viruses per week by total number of viruses in that subtype/lineage) of viruses by assigned clade by subtype (A, A(H1N1)pdm09; B, A(H3N2); C, B/Victoria) and week, EU/EEA, weeks 40/2022- 39/2023.

A. A(H1N1)pdm09

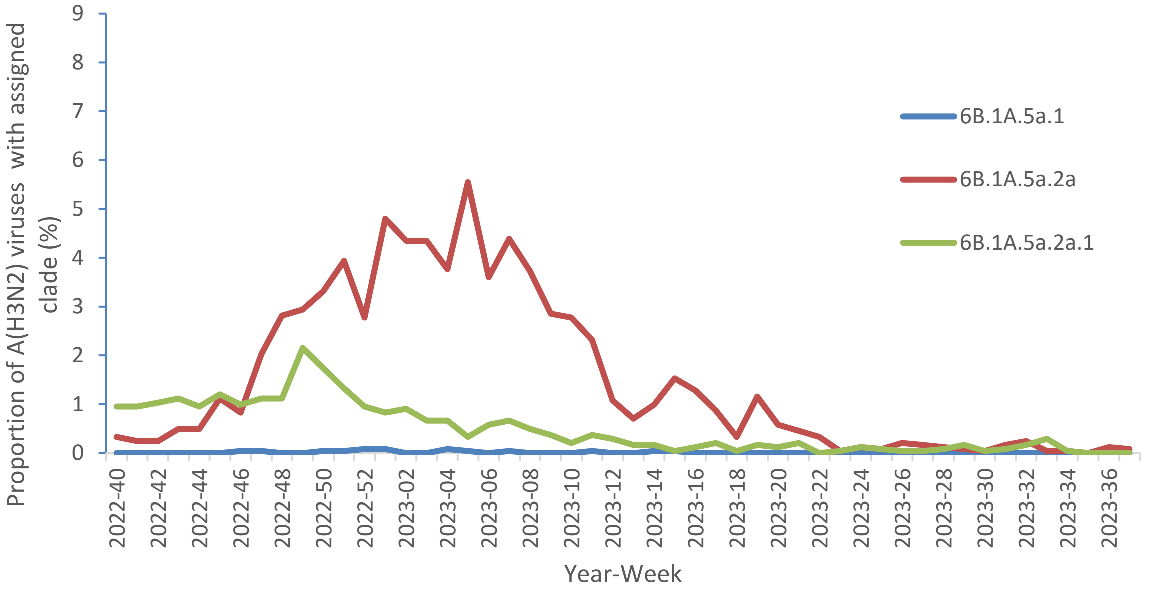

B. A(H3N2)

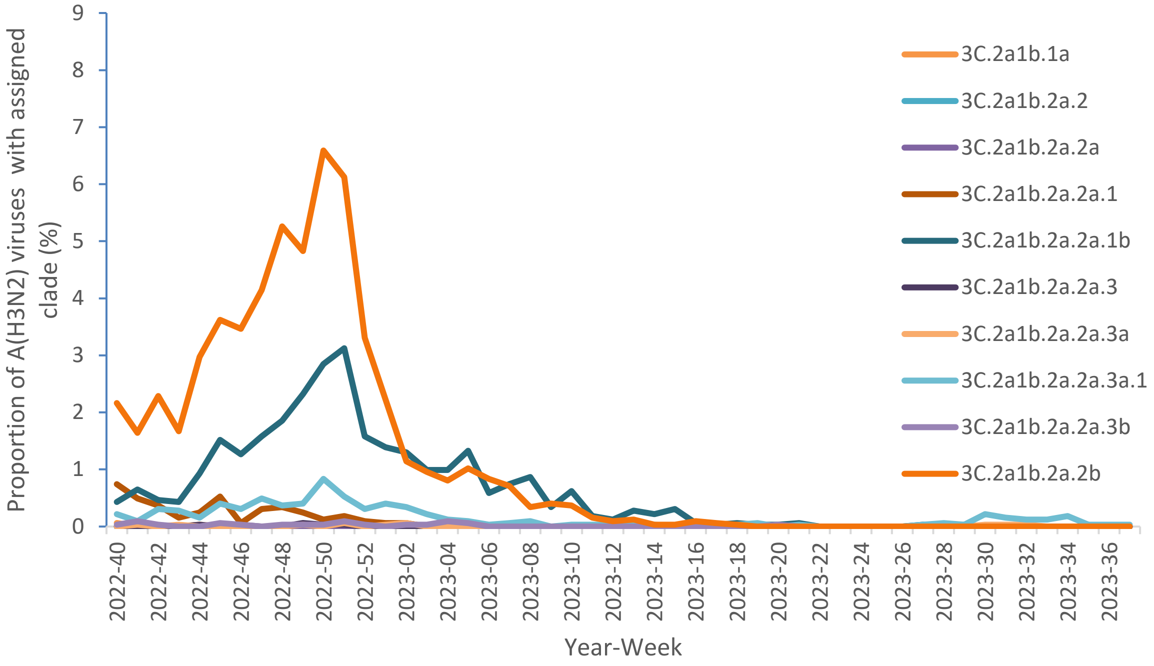

C. B/Victoria

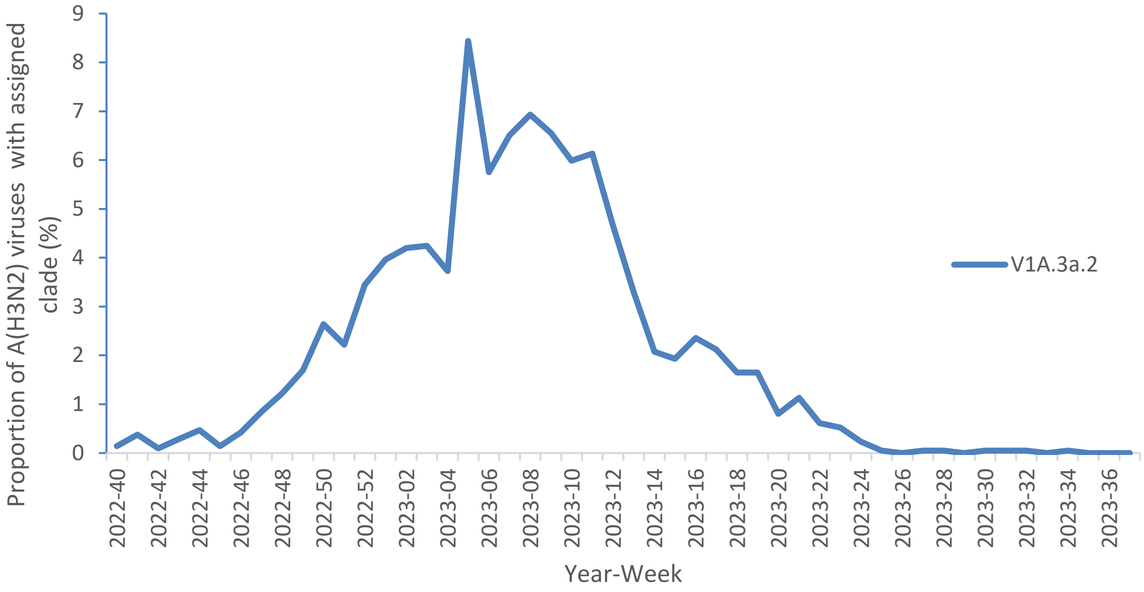

Proportion of A(H3N2) viruses with assigned clade (%)
9
8
7
6
5
4
3
2
1
0
2022-40
2022-42
2022-44
2022-46
2022-48
2022-50
2022-52
2023-02
2023-04
2023-06
2023-08
2023-10
2023-12
2023-14
2023-16
2023-18
2023-20
2023-22
2023-24
2023-26
2023-28
2023-30
2023-32
2023-34
2023-36
Year-Week
V1A.3a.2

Supplemental figure SF3: Number of antigenic and genetic clade categoric reports and number of countries reporting each type of data to TESSy, by influenza season, EU/EEA countries, 2016-2023.

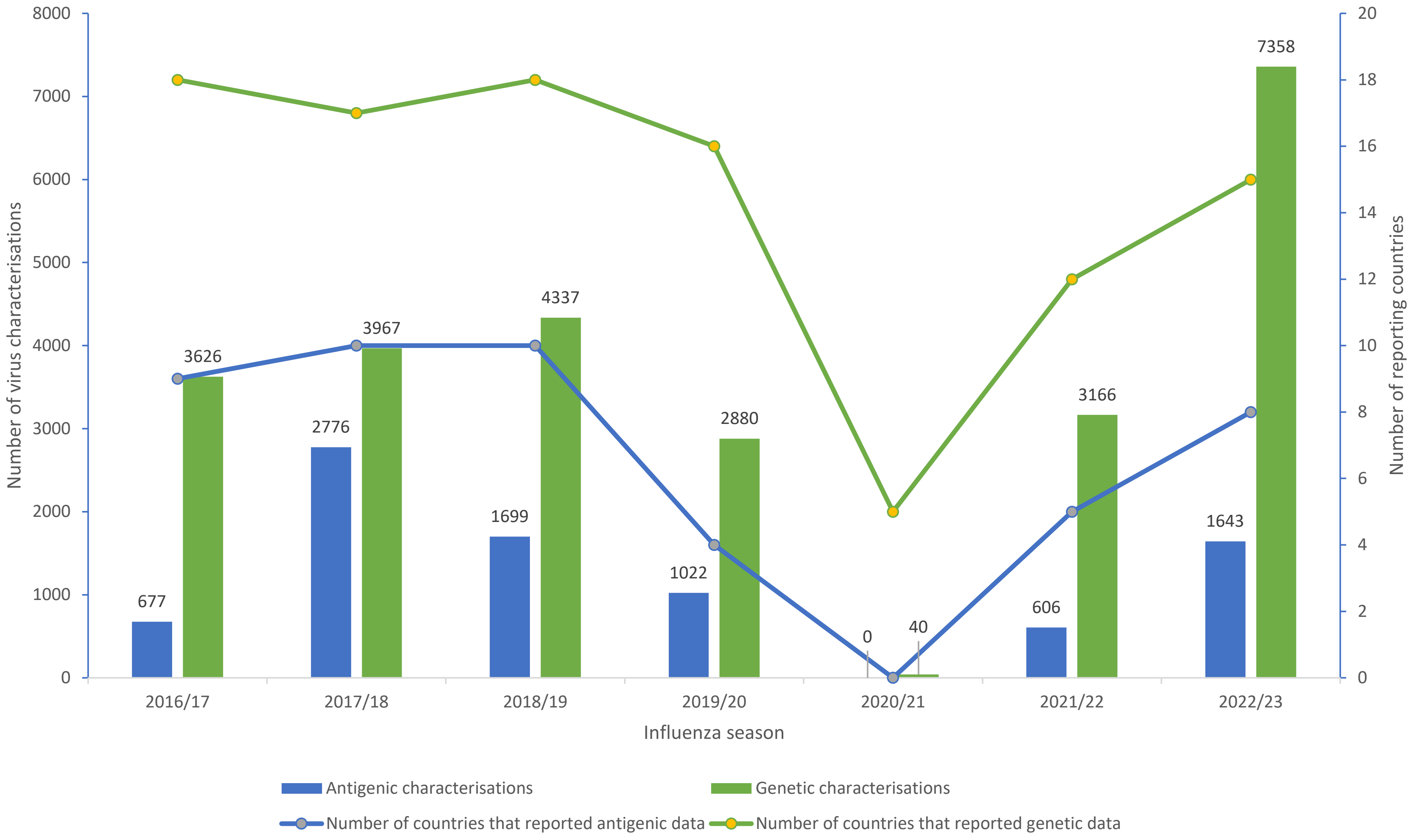

Broberg et al.
